# Supplementary material for: Monitoring the reproductive number of COVID-19 in France: Comparative estimates from three datasets
Source: PLoS One. 2023 Oct 31;18(10):e0293585. doi: 10.1371/journal.pone.0293585 (PMC10617725; doi:10.1371/journal.pone.0293585)
Supplement: S1 File — (DOCX) [file pone.0293585.s001.docx]

**Supporting information: Details on the Cori’s method for estimating effective reproduction numbers**

**Monitoring the reproductive number of COVID-19 in France: Comparative estimates from three datasets**

Christophe Bonaldi (1), Anne Fouillet (1), Cécile Sommen (1), Daniel Lévy-Bruhl (2), Juliette Paireau (2)(3)

(1) Data Science Division, Santé publique France, the French Public Health Agency, Saint Maurice, France

(2) Infectious Diseases Division, Santé publique France, the French Public Health Agency, Saint Maurice, France

(3) Mathematical Modelling of Infectious Diseases Unit, Institut Pasteur, Université de Paris Cité, CNRS UMR 2000, Paris, France

**S1: How do the parameters of the serial interval distribution affect estimates of the number of reproductions?**

The mean and the standard deviation of the serial interval distribution (gamma distribution [1,2]) has an impact on the value of the effective reproduction number (R*t*) estimates, but do not affect the temporal shape. In the following figure, we show this shape for the estimates using the SI-VIC data (May 27, 2020, to August 12, 2022) for 3 values of the mean for the gamma distribution: 3, 5.2 and 7 days with a standard deviation of 4.7 days. We see that R*t* estimates become higher as the mean of the serial interval increases.


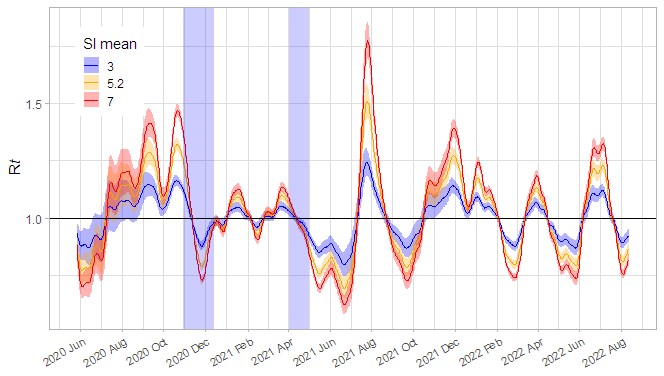


In addition, the higher the standard deviation of the serial interval, the lower the Rt estimates [3]. For example, with 7 days for the mean of the gamma distribution and 0.2, 3.2and 6.2 days for the standard deviation, we get the following curves:


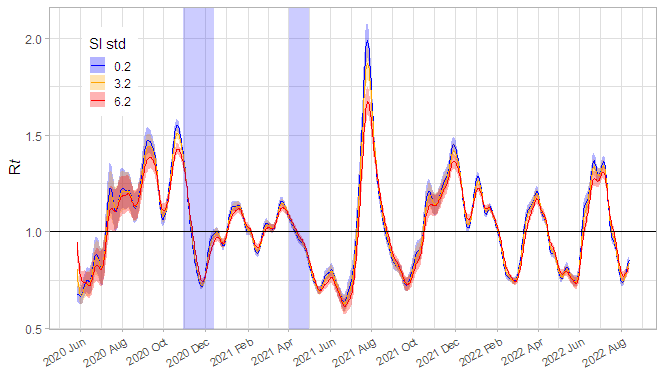


**S2: Cori’s method to estimate the effective reproduction number: formal framework (parametric version)**

Let the number of newly infected cases on day *t* be $I_{t}$. Following Fraser [4], conditionnaly to a serial interval distribution $w_{s}$, the total number of infected cases up to time *t* and the time-dependent reproduction number R*t,* the expected number of newly infected cases at time *t,* is :

$$E\left( {I_{t}}/{I_{0},\ldots, I_{t-1},w_{s},Rt} \right)=Rt\Lambda_{t}\left( w_{s} \right)$$

$\Lambda_{t}\left( w_{s} \right)= \sum_{s=1}^{t} I_{t-s} w_{s}$ is the total infection potential across all infected cases at time *t* where $I_{t-s} w_{s}$ represent the contribution of the $I_{t-s}$ infected cases at time $t-s$ to secondary cases arising after a time period ***s.***

Assuming that the number of incident cases at time *t* is drawn from a Poisson distribution, the conditionnal probability of observing $I_{t}$ is :

$$P\left( {I_{t}}/{I_{0},\ldots, I_{t-1},w_{s},Rt} \right)=\frac{\left( Rt\Lambda_{t}\left( w_{s} \right) \right)^{I_{t}}}{I_{t}!}e^{-Rt\Lambda_{t}\left( w_{s} \right)}$$

In addition, to stabilise the variance of the estimates and to facilitate interpretation, it is assumed that the reproduction number is constant over a time period $\left[ t-\tau, t \right]$ and then :

$$P\left( {I_{t-\tau},\ldots,I_{t}}/{I_{0},\ldots, I_{t-\tau-1},w_{s},Rt} \right)=\prod_{k=t-\tau}^{t} \frac{\left( Rt\Lambda_{k}\left( w_{s} \right) \right)^{I_{t}}}{I_{k}!}e^{-Rt\Lambda_{k}\left( w_{s} \right)}$$

Using Bayes’ formula $P\left( \frac{A}{B} \right)=\frac{P\left( \frac{B}{A} \right)P\left( A \right)}{P\left( B \right)}$ with $\left\{ \begin{aligned} A=R_{t} \\ B= \left( \frac{I_{t-\tau},\ldots,I_{t}}{I_{0},\ldots,I_{t-\tau-1},w_{s}} \right) \end{aligned} \right.$, this give us the posterior distribution for R*t* :

$$P\left( {Rt}/{I_{0},\ldots, I_{t},w_{s}} \right)\propto P\left( \frac{I_{t-\tau},\ldots, I_{t}}{I_{0},\ldots,I_{t-\tau-1},w_{s},Rt} \right) P\left( Rt \right)$$

Under the assumption that the prior distribution of R*t* is a Gamma distribution with shape *a* and scale *b* parameters, thus:

$$P\left( {Rt}/{I_{0},\ldots, I_{t},w_{s}} \right)\propto\left( \prod_{k=t-\tau}^{t} \frac{\left( Rt\Lambda_{k}\left( w_{s} \right) \right)^{I_{k}}}{I_{k}!}e^{{-R}_{t}\Lambda_{k}\left( w_{s} \right)} \right)\left( \frac{{Rt}^{a-1}e^{\left( -\frac{Rt}{b} \right)}}{\Gamma\left( a \right)b^{a}} \right)$$

$$\propto{Rt}^{a+\sum_{k=t-\tau}^{t} I_{k}-1}e^{-Rt\left( \sum_{k=t-\tau}^{t} \Lambda_{k}\left( w_{s} \right)+\frac{1}{b} \right)}\prod_{k=t-\tau}^{t} \frac{{\Lambda_{k}\left( w_{s} \right)}^{I_{k}}}{I_{k}!}$$

According to the expression above and using the following new parametrization:

$$\left\{ \begin{aligned} K= a+\sum_{k=t-\tau}^{t} I_{k} \\ \Theta=\left( \sum_{k=t-\tau}^{t} \Lambda_{k}\left( w_{s} \right)+\frac{1}{b} \right)^{-1} \end{aligned} \right.$$

We can write:

$${Rt}^{a+\sum_{k=t-\tau}^{t} I_{k}-1}e^{-Rt\left( \sum_{k=t-\tau}^{t} \Lambda_{k}\left( w_{s} \right)+\frac{1}{b} \right)}={Rt}^{K-1}e^{\left( -\frac{\mathbf{R}\boldsymbol{t}}{\Theta} \right)}$$

To within a constant $\left( \frac{1}{\Gamma\left( K \right)\Theta^{K}} \right)$, we recognise a Gamma distribution with shape $K$ and scale $\Theta$ parameters. The posterior mean of R*t* is straightforwardly deduced:

$$E\left( {R_{t}}/{I_{0},\ldots, I_{t},w_{s}} \right)=\frac{K}{\Theta} =\frac{a+\sum_{k=t-\tau}^{t} I_{k}}{\left( \sum_{k=t-\tau}^{t} \Lambda_{k}\left( w_{s} \right)+\frac{1}{b} \right)^{-1}}$$

and 95%confidence interval are provided by the 2.5% and 97.5% quantiles of the Gamma distribution with parameters $\left( K,\Theta\right)$.

It should be noted that in our analyses, we keep a prior relatively uninformative for R*t* with parameters *a* and *b* such that the mean and the standard deviation equal to 5, as in Thompson *et al.* [1] or Cori *et al.* [2] (the default prior for R*t* in *EpiEstim* package).

**References**

1. Thompson RNN, Stockwin JEE, van Gaalen RDD, Polonsky JAA, Kamvar ZNN, Demarsh PAA, et al. Improved inference of time-varying reproduction numbers during infectious disease outbreaks. Epidemics. 2019;29: 100356. PMID:31624039

2. Cori A, Ferguson NM, Fraser C, Cauchemez S. A new framework and software to estimate time-varying reproduction numbers during epidemics. Am J Epidemiol. 2013;178: 1505–12. PMID:24043437

3. Wallinga J, Lipsitch M. How generation intervals shape the relationship between growth rates and reproductive numbers. Proceedings Biol Sci. 2007;274: 599–604. PMID:17476782

4. Fraser C. Estimating Individual and Household Reproduction Numbers in an Emerging Epidemic. Galvani A, editor. PLoS One. 2007;2: e758. PMID:17712406

The code and data for estimating R*t* provided in the paper are available from the GitHub repository (<https://github.com/christophe-bonaldi/Monitoring-R-effective>)
